# Molecular Characterization of Constipation Disease as Novel Phenotypes in CRISPR-Cas9-Generated Leptin Knockout Mice with Obesity

**DOI:** 10.3390/ijms21249464

**Published:** 2020-12-12

**Authors:** Ji Eun Kim, Yun Ju Choi, Su Jin Lee, Jeong Eun Gong, Yong Lim, Jin Tae Hong, Dae Youn Hwang

**Affiliations:** 1 Department of Biomaterials Science (BK21 FOUR Program), College of Natural Resources & Life Science, Life and Industry Convergence Research Institute, Laboratory Animal Resources Center, Pusan National University, Miryang 50463, Korea; prettyjiunx@naver.com (J.E.K.); poiu335@naver.com (Y.J.C.); nuit4510@naver.com (S.J.L.); jegog@naver.com (J.E.G.); 2 Department of Clinical Laboratory Science, College of Nursing and Healthcare Science, Dong-Eui University, Busan 47340, Korea; yonglim@deu.ac.kr; 3 College of Pharmacy, Chungbuk National University, Chungju 28160, Korea; jinthong@chungbuk.ac.kr

**Keywords:** constipation, leptin, knockout mice, gastrointestinal motility, muscle contraction, mAChR

## Abstract

(1) Background: We characterized a novel animal model with obesity-induced constipation because constipation is rarely known in genetically engineered mice (GEM); (2) Methods: The changes in the constipation parameters and mechanisms were analyzed in CRISPR-Cas9-mediated leptin (Lep) knockout (KO) mice from eight to 24 weeks; (3) Results: Significant constipation phenotypes were observed in the Lep KO mice since 16 weeks old. These mice showed a significant decrease in the gastrointestinal motility, mucosal layer thickness and ability for mucin secretion as well as the abnormal ultrastructure of Lieberkühn crypts in the transverse colon. The density or function of the enteric neurons, intestinal Cajal cells (ICC), smooth muscle cells, and the concentration of gastrointestinal (GI) hormones for the GI motility were remarkably changed in Lep KO mice. The downstream signaling pathway of muscarinic acetylcholine receptors (mAChRs) were activated in Lep KO mice, while the expression of adipogenesis-regulating genes were alternatively reduced in the transverse colon of the same mice; (4) Conclusions: These results provide the first strong evidence that Lep KO mice can represent constipation successfully through dysregulation of the GI motility mediated by myenteric neurons, ICC, and smooth muscle cells in the transverse colon during an abnormal function of the lipid metabolism.

## 1. Introduction

Constipation involves infrequent bowel movements or difficult passage of stools characterized by hard and dry stools, incomplete bowel evacuation, and difficulty during defecation [1,2,3]. The action mechanism and the efficacy of drugs for this disease have been examined in various animal models with constipation phenotypes for a long time. However, most are generated by administering chemical compounds, such as loperamide and carbon, and only a few were observed in a few GEMs, including transgenic (Tg) mice and KO mice [4,5,6,7]. Among these, BDNF^+/-^ mice showed significant decreases in the stool frequency, water contents, total gastrointestinal transit, protein gene product 9.5 (PGP9.5) expression for neural marker and α-smooth muscle actin (α-SMA) expression for a smooth muscle marker [6], while the Pitt–Hopkins syndrome (PTHS) mouse model show a decrease in upper gastrointestinal transit and distal colon transit velocities [8]. In addition, the intestinal length, thickness of the intestinal smooth muscle, and intestinal contractibility were significantly lower in SMC-specific Nrp1 KO mice than those of wild-type mice [9]. Similar phenotypes, including an increase in stool retention and a decrease in pellet number, stool weight, and water content was observed in adenosine 2B receptor (A2BAR) KO mice and tenascin-X KO mice [10,11]. Various constipation phenotypes, including stool parameters, histopathological structure, and mucin secretion, as well as their molecular mechanisms, such as mAChR signaling and endoplasmic reticulum (ER) stress response, were detected in Tg2576 Tg mice showing Alzheimer’s Disease (AD)-like phenotypes [7]. On the other hand, no significant scientific evidence has been provided to verify the constipation phenotypes in the GEM model with obesity although these phenotypes and mechanism have been studies in a diet-induced obese model and clinically obese patients [12,13].

The *ob/ob* mouse model is the most popular GEM model in obesity research. This model was first established at the Jackson Laboratory in 1949 as a mutant mouse that overeats and becomes obese in a colony [14]. This mutation was identified as a single autosomal recessive mutation on the obese gene (leptin) by positional cloning [15,16]. This *ob/ob* mouse represents various symptoms, including obesity, hyperglycemia, hyperinsulinemia, enlargement of pancreatic islets, and insulin resistance [17]. Based on the above properties, *ob/ob* mice have been used to evaluate many of the same drugs and compounds, including anti-hyperglycemic agents, thiazolidinediones (TZDs), and TZDs derivatives [18,19,20]. CRISPR-Cas9-induced Lep KO mice (C57BL/6-Lep^em1Shwl^/Korl) was produced as an obese model by the co-microinjection of Cas9 mRNA and sgRNAs in C57BL/6J embryos recently. The mice showed phenotypic disorders, such as increased body weight, hyperglycemia, and hepatic steatosis [21]. However, the association of chronic constipation with obesity in Lep KO mice has not been confirmed, even though constipation was observed in 8.3% of obese patients [12,22,23].

This study examined the scientific evidence for the pathological symptoms and molecular mechanism of constipation in Lep KO mice through an analysis of the stool parameters, histopathology, GI transit, mucin secretion, myenteric neuron function, smooth muscle contraction, and lipid metabolism during obesity. These results suggest that Lep KO mice can be considered novel animal models for obesity-induced constipation with dysregulation on the GI motility and smooth muscle contraction.

## 2. Results

### 2.1. Confirmation of Obesity Phenotypes in Lep KO Mice

**Figure 1:**
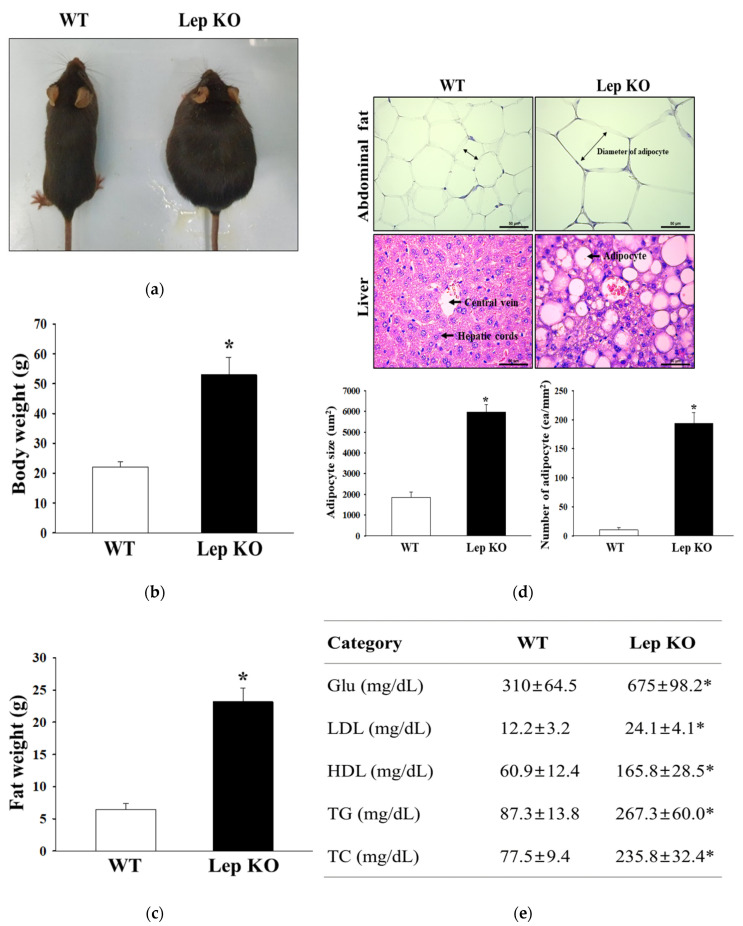
Obesity phenotypes of Lep KO mice. (**a**) Morphology of WT and Lep KO mice was observed at 24 weeks of age; (**b**) Body weights of the mice of the subset group were measured, as described in Materials and Methods. Five to six mice per group were selected from each group, and their weights were assayed in duplicate for each sample; (**c**) After collecting the retroperitoneal fat, their weights were measured using an electric balance. Five to six mice per group were used to collect the fat tissues; the weight of fat tissue was measured in duplicate for each tissue; (**d**) After hematoxylin and eosin (H&E) staining of the fat and liver tissue, their histopathological features were observed at 100× magnification using an optical microscope. The area of each adipocyte in fat and lipid droplets in the liver was measured using the Image J program. Five to six mice per group were used in histological analysis, and each parameter was measured in duplicate in two different slides; (**e**) Serum levels of Glu, LDL-C, HDL-C, TG, and TC were determined in all mice of the subset groups. Five to six mice per group were used in the preparation of serum. Biochemical analysis was assayed in duplicate for each sample. Data are reported as the mean ± SD. * indicates *p* < 0.05 compared to WT mice. Abbreviations: M, Marker; WT, Wild type; KO, Knockout type; TC, total cholesterol; TG, triglyceride; LDL-C, low-density lipoprotein–cholesterol; HDL-C, high-density lipoprotein–cholesterol; Glu, glucose.

The key indicators for the pathological phenotypes of obesity, including an increase in body weight, hyperglycemia, hepatic steatosis and adiposity, have been reported in the Lep KO mice [21]. Therefore, to confirm the successful induction of the obesity phenotype, body weight, fat accumulation and serum lipid profiles in Lep KO mice were analyzed. The increase in body weight was dependent on their age from eight to 24 weeks (Figure S1a). At 24 weeks, the body weight and weight of the retroperitoneal fat were 1.62 and 2.54 times higher in the Lep KO mice, respectively, than the wild type (WT) mice, but the increase rate was greater in the fat weight (Figure 1a–c). Significant changes were detected in the hematoxylin and eosin (H&E)-stained fat and liver sections. The adipocyte size in fat tissue increased two-fold in the Lep KO mice compared to the WT mice although their number was constantly maintained. However, the number of lipid droplets in the liver tissue increased 18-fold in the same group (Figure 1d). A similar increase pattern in Lep KO mice was observed in the lipid profile, including glucose (Glu), low-density lipoprotein–cholesterol (LDL-C), high-density lipoprotein–cholesterol (HDL-C), triglyceride (TG), and total cholesterol (TC) (Figure 1e). These results suggest that the Lep KO mice used in the current study showed the prominent pathological phenotypes for obesity.

### 2.2. Alterations on the Feeding Behavior and Stool Parameters of Lep KO Mice

**Figure 2:**
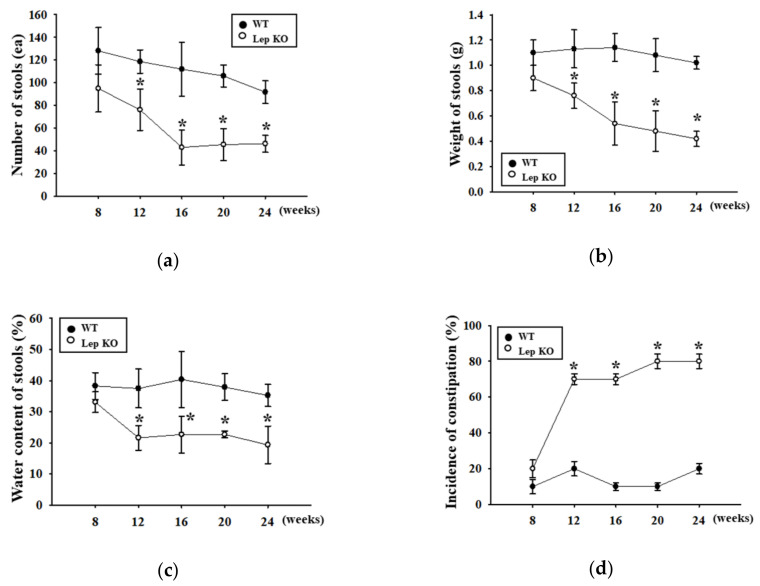

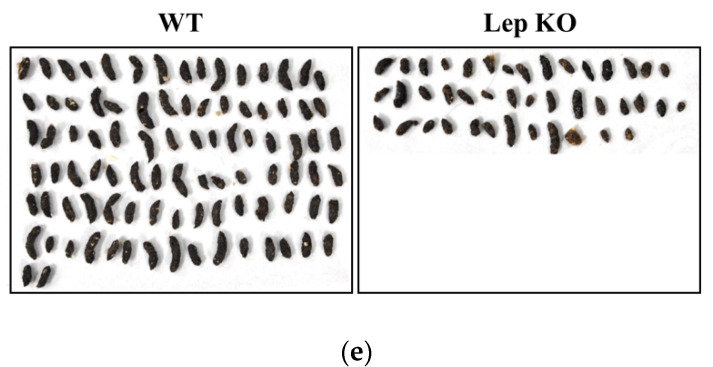
Incidence of constipation. The number (**a**), weight (**b**), and water contents (**c**) of the stool were measured from eight to 24 weeks, as described in the Materials and Methods; (**d**) The incidence of constipation was calculated based on the number of constipated mice and total mice used during 16 weeks, as described in Materials and methods; (**e**) The morphology of stools was observed using a digital camera. Five to six mice per group were used for the stool sample collection, and each parameter was assayed in duplicate. The data are reported as the mean ± SD. * indicates *p* < 0.05 compared to the WT mice. Abbreviations: WT, Wild type; KO, Knockout type.

The changes in the stool parameters, food intake, and water consumption of Lep KO mice during obesity were measured to determine if a Lep-deficiency-induced obesity might be associated with the constipation phenotypes. The stool parameters, including the number, weight, and water content, were decreased gradually in the Lep KO mice from eight weeks to 24 weeks compared to the WT mice. The significant constipation phenotype, including the decrease in stool number, weight, and water contents, was first detected in Lep KO mice at 12 weeks (Figure 2a–c). At this time, the measured incidence of constipation was 70%, which increased slightly to 80% from 16 to 24 weeks (Figure 2d). At 24 weeks, the Lep KO mice showed a 49.6%, 58.8%, and 45.1% decrease in the number, weight, and water content of stools, respectively, compared to the WT mice (Figure 2a–c). The normal morphology of the stools changed to an irregular and small shape in the Lep KO mice (Figure 2e). However, food intake and water consumption were constant in the same groups (Figure S1b). These results suggest that defecation delay can be induced successfully during Lep deficiency-induced obesity in Lep KO mice without significant changes in their feeding behavior.

### 2.3. Alterations on the Gastrointestinal Motility and Intestinal Length of Lep KO Mice

**Figure 3:**
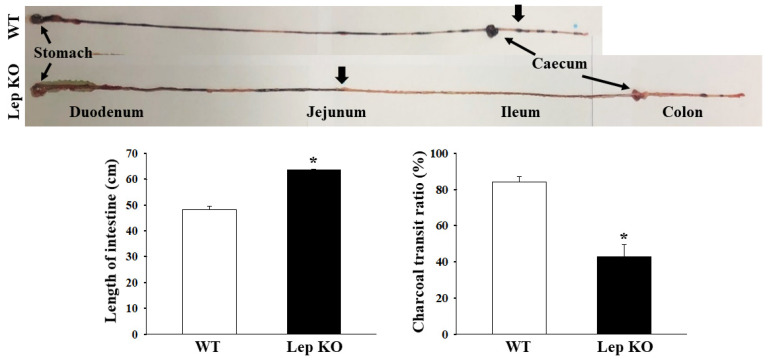
GI transit ratio and intestinal length of Lep KO mice. The total intestinal tract was excised from the rats of the subset groups treated with charcoal meal powder. The morphology was observed using a digital camera. The arrows indicate the position of the charcoal meal. The total distance traveled by the charcoal meal from the pylorus was measured. The charcoal meal transit ratio was then calculated using the total length of the intestine and the distance of the charcoal meal. Three to five mice per group were used in the gastrointestinal transit ratio test, and the charcoal meal transit distance and intestine length were measured in duplicate. The data are reported as the mean ± SD. * indicates *p* < 0.05 compared to the WT mice. Abbreviations: WT, Wild type; KO, Knockout type.

A charcoal meal transit test [24] and intestine length analyses were performed in Lep KO mice at 24 weeks of age to determine if the defecation delay in Lep KO mice are accompanied by alterations in the gastrointestinal motility and intestinal length. The propulsion of the charcoal meal decreased by 50% in the Lep KO mice compared to the WT mice. On the other hand, a reverse pattern was observed in the intestine length; the length was 33.3% longer in the Lep KO mice than in the WT mice (Figure 3). These results suggest that the defecation delay in Lep KO mice is associated with a dysregulation of the gastrointestinal motility and an increase in intestinal length. 

### 2.4. Alterations in the Histopathological and Cytological Structure in the Transverse Colon of Lep KO Mice

**Figure 4:**
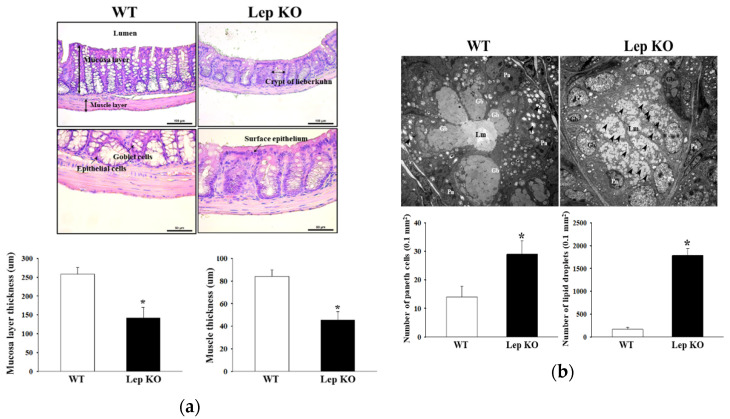
Histological structures and ultrastructure of the transverse colon. (**a**) The hematoxylin and eosin (H&E)-stained sections of the transverse colon from the WT and Lep KO mice were observed at 100× and 400× magnification using an optical microscope. The histopathological parameters were determined using the Leica Application Suite (Leica Microsystems). Three to five mice per group were used for histological analysis, and each parameter was measured in duplicate in two different slides; (**b**) The crypt ultrastructure of the transverse colon in the WT and Lep KO mice was observed by TEM at 4000× magnification. The number and size of goblet cells and the number of mucin droplets (arrowhead) per each goblet cell were determined using Leica Application Suite software. Two to three mice per group were used to prepare the TEM slide, and three parameters were assayed in duplicate in each test. The data are reported as the mean ± SD. * indicates *p* < 0.05 compared to the WT mice. Abbreviations: WT, Wild type; KO, Knockout type; Pn, Paneth cells; Gb, Goblet cells; Lm, Lumen.

The changes in the histological parameters, indicating constipation in the H&E-stained transverse colons of KO and WT mice, were examined to determine the associated changes in the histopathological structure of the transverse colon caused by the defecation delay in Lep KO mice. The thicknesses of the mucosa layer and muscle of the transverse colon were significantly lower in the Lep KO mice than the WT mice (Figure 4a). Significant changes were also observed in the crypts of Lieberkühn in the transverse colon sections of Lep KO mice (Figure 4a). The structure of the crypts changed dramatically from the open form to the luminal face in WT mice to a distinct elliptical form in the Lep KO mice. In particular, the irregular shape, distribution, and size of goblet cells in the crypts were detected in the H&E-stained section of the transverse colon in Lep KO mice (Figure 4a). In addition, the associated changes in the ultrastructure of crypts were examined by transmission electron microscopy (TEM). Significant alterations were observed on the crypts of Lieberkühn of the transverse colon. The number of goblet cells was higher in the Lep KO mice than the WT mice, but the average size and average number of mucin drops in each goblet cell were lower in the same mice. Similar increases in the number of epithelial cells were observed (Figure 4b). These findings show that defecation delay effects in Lep KO mice are associated with the abnormalities in the histopathological and cytological structural of the transverse colon.

### 2.5. Alterations on the Mucin Secretion Ability and Membrane Water Channel Expression in the Transverse Colon of Lep KO Mice

**Figure 5:**
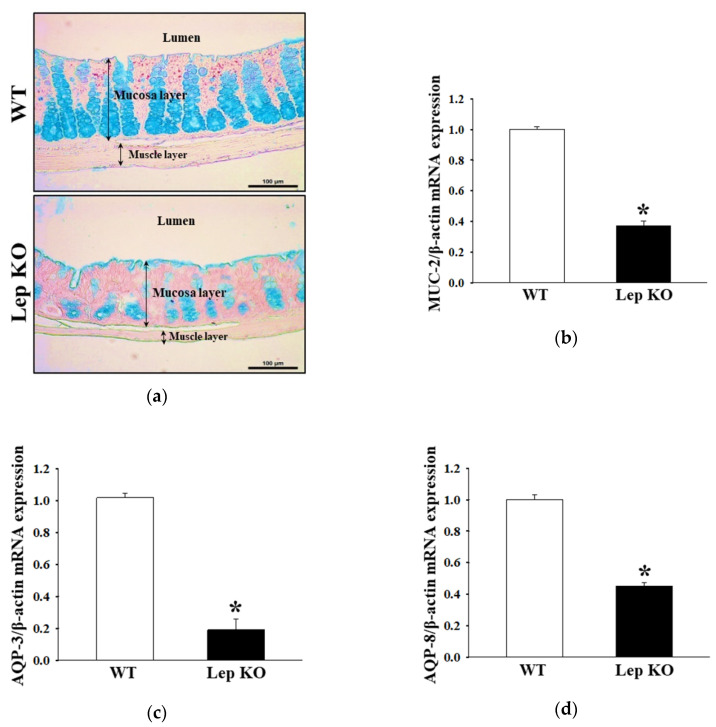
Mucin secretion and membrane water channel expression. (**a**) Mucin secreted from crypt layer cells was stained with alcian blue at pH 2.5. Images were observed at 400× magnification. Five to six mice per group were used in the slide section, and mucin staining was assessed in duplicate in two different slides. The levels of MUC2 (**b**), AQP3 (**c**), and AQP8 (**d**) transcripts in the total mRNA of transverse colons were measured by RT-qPCR using the specific primers. The mRNA levels of three genes were calculated based on the transcript level of β-actin as an endogenous control. Three to five mice per group were used to prepare the total RNA, RT-qPCR analyses were assayed in duplicate for each sample. The data are reported as the mean ± SD. *, *p* < 0.05 compared to the WT group. Abbreviations: WT, Wild type; KO, Knockout type; RT-qPCR, Quantitative real time-PCR; MUC2, Mucin 2; AQP, Aquaporin.

Experiments were performed to determine if the defecation delays in Lep KO mice are accompanied by changes in the regulation of mucin secretion ability and water transport in the transverse colon. The levels of mucin secretion and membrane water channel expressions were measured in the transverse colon of Lep KO and WT mice. The goblet cells stained with dark blue for mucin were concentrated in the crypts of Lieberkühn of WT mice (Figure 5a). On the other hand, this intensity decreased remarkably in the transverse colon of Lep KO mice compared to WT mice (Figure 5a). These alterations were completely reflected in the level of the mucin 2 (MUC2) transcript (Figure 5b). Furthermore, a similar regulation pattern in the levels of the aquaporin (AQP)3 and AQP8 transcripts was observed. These levels were 81.3% and 54.8% lower in the Lep KO mice than the WT mice (Figure 5c,d). These results show that the defecation delay in Lep KO mice were associated with the decreasing ability to secrete mucin and expression of a membrane water channel in the transverse colon.

### 2.6. Alterations on the Loss of Nitrergic Enteric and Myenteric Neurons in the Transverse Colon of Lep KO Mice

**Figure 6:**
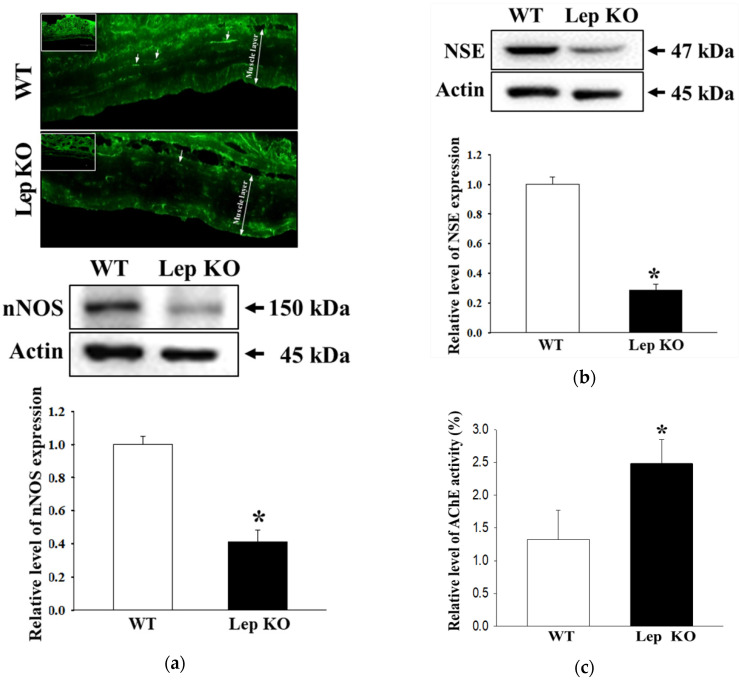
Expression of nNOS and NSE, and AChE activity. (**a**) The tissue distribution of nNOS was detected in the transverse colon using immunofluorescence (IF) staining assay. Three to five mice per group were used in the slide section, and IF staining was assessed in duplicate in two different slides. The expression levels of nNOS were measured by Western blot analysis using the specific primary antibodies and HRP-labeled anti-rabbit IgG antibody. Three to five mice per group were used to prepare the total tissue homogenate, and Western blot analyses were assayed in duplicate in each sample; (**b**) The expression levels of NSE were measured by Western blot analysis using the specific primary antibodies and HRP-labeled anti-rabbit IgG antibody. Three to five mice per group were used to prepare the total tissue homogenate, and Western blot analyses were assayed in duplicate in each sample; (**c**) After homogenizing the transverse colon tissue, the AChE activity was measured using an Acetylcholinesterase Assay Kit that could detect as little as 0.01 mU AChE in a 100 μL assay volume (0.1 mU/mL). Three to five mice per group were used to prepare the tissue lysate, and enzyme activity was assayed in duplicate in each test. The data are reported as the mean ± SD. *, *p* < 0.05 compared to the WT group. Abbreviations: WT, Wild type; KO, Knockout type; nNOS, Neuronal nitric oxide synthase; NSE, Neuron-specific enolase; AChE, Acetylcholinesterase.

We examined whether the defecation delay in Lep KO mice are accompanied by alterations in the density and function of nitrergic enteric and myenteric neurons in the transverse colon. First, to examine the effects on the density of nitrergic enteric neurons, the distribution of neuronal nitric oxide synthase (nNOS)-stained cells and levels of nNOS expression were measured in the transverse colon of Lep KO mice because nNOS was the predominate form in enteric neurons [25,26]. Morphometric analysis of the neural population showed a lower nNOS-stained subpopulation in the smooth muscle layer of the transverse colon in Lep KO mice than the WT mice. A similar pattern was also observed in the expression level of nNOS according to Western blot analysis (Figure 6a). These results indicate that the defecation delay in the Lep KO mice may be associated with the loss of nitrergic enteric neurons, which are key players in the descending inhibitory reflex of intestinal peristalsis [25,26].

The changes in the expression level of neuron-specific enolase (NSE) and acetylcholinesterase (AChE) activity in the transverse colon of Lep KO mice were measured to examine the density and functional activity of myenteric neurons in the transverse colon. The levels of NSE expression were 75% lower in the Lep KO mice than the WT mice, whereas the AChE activity was 92% higher in the Lep KO mice than WT mice (Figure 6b,c). These results suggest that the defecation delay in Lep KO mice may be associated with the loss of myenteric neurons and an increase in AChE activity in the transverse colon.

### 2.7. Alterations on the Smooth Muscle Contraction in the Transverse Colon of Lep KO Mice

**Figure 7:**
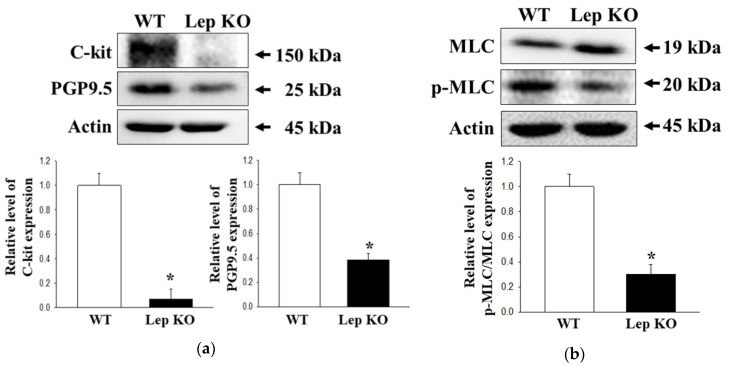

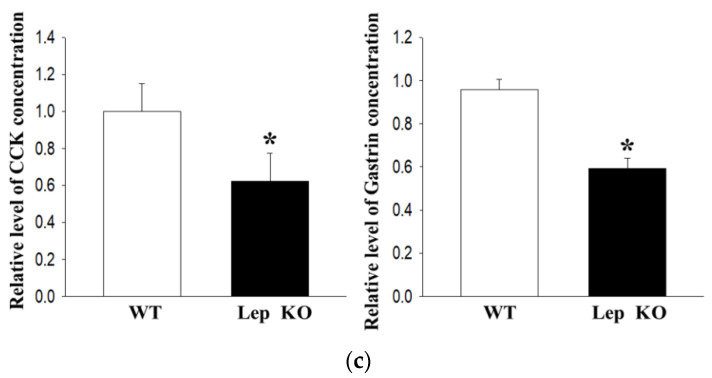
Expression of C-kit and PGP9.5, phosphorylation of MLC, and serum concentration of GI hormones. (**a**) Expression levels of C-kit and PGP9.5 were measured by Western blot analysis using specific primary antibodies and HRP-labeled anti-rabbit IgG antibody. Three to five mice per group were used to prepare the total tissue homogenate, and Western blot analyses were assayed in duplicate in each sample; (**b**) Expression levels of MLC and p-MLC were measured by Western blot analysis using the specific primary antibodies and HRP-labeled anti-rabbit IgG antibody. After determining the intensity of each band using an imaging densitometer, the relative levels of the four proteins were calculated based on the intensity of actin. Three to five mice per group were used to prepare the total tissue homogenate, and Western blot analyses were assayed in duplicate in each sample; (**c**) The concentrations of CCK and gastrin were measured in the transverse colon homogenate by an enzyme-linked immunosorbent assay. The minimum detectable concentration of each kit was 0.1–1000 pg/mL of CCK and 0.312–20 pg/mL of gastrin. Three to five mice per group were used to prepare the tissue homogenate, and the hormone level was assayed in duplicate in each sample. The data are reported as the mean ± SD. *, *p* < 0.05 compared to the WT group. Abbreviations: WT, Wild type; KO, Knockout type; C-kit, Receptor tyrosine kinase; PGP9.5, Protein gene product 9.5; MLC, Myosin light chain; GI, Gastrointestinal; CCK, Cholecystokinin.

The smooth muscle contraction of the intestine is a highly interactive process, including intestinal Cajal cells (ICC), smooth muscle cell, and GI hormone [27,28,29]. To determine if the defecation delay in Lep KO mice are accompanied by changes in the smooth muscle contraction of transverse colon, alterations in the expression level of ICC markers (receptor tyrosine kinase (C-kit) and PGP9.5), smooth muscle cells marker (myosin light chain (MLC)), and concentration of GI hormone were measured in the transverse colon of Lep KO mice. The levels of C-kit and PGP9.5 expressions were lower in the transverse colon of Lep KO mice compared to the WT mice (Figure 7a). In addition, a similar decrease in Lep KO mice was detected in the phosphorylation of MLC, but the rate of the decrease was different (Figure 7b). Furthermore, the concentrations of cholecystokinin (CCK) and gastrin were lower in the transverse colon of Lep KO mice than the WT mice (Figure 7c). These results suggest that the defecation delay in Lep KO mice might be associated with the suppression of smooth muscle contractions through the dysregulation of ICC, smooth muscle cells, and GI hormone.

### 2.8. Alterations on the Downstream Signaling Pathway of mAChRs in the Transverse Colon of Lep KO Mice

**Figure 8:**
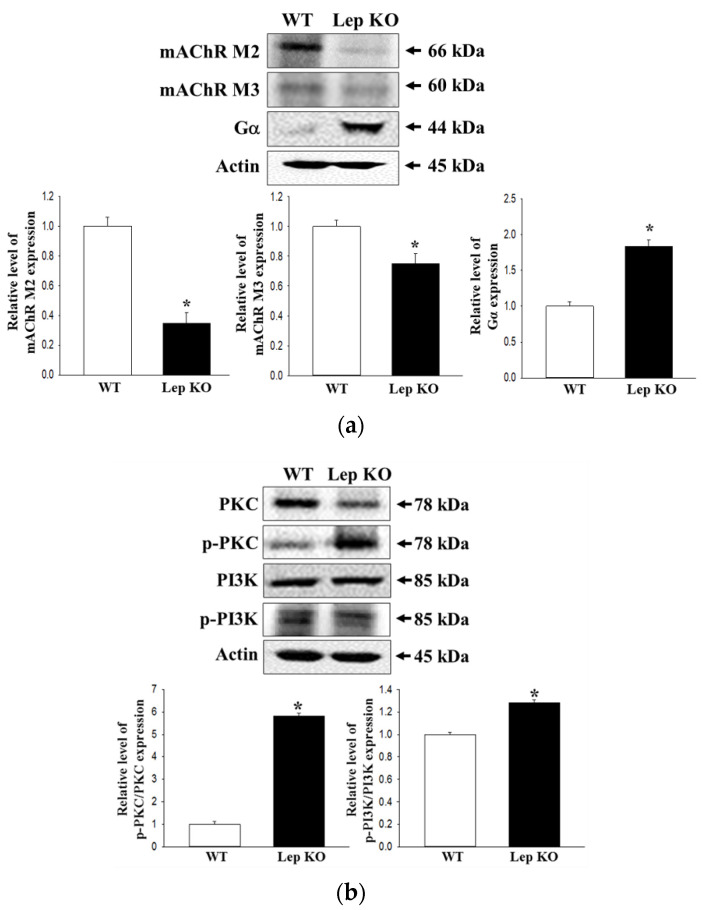
Expression of mAChRs and key mediators within their downstream signaling pathway. (**a**) Expression levels of mAChR M2 and mAChR M3 were measured by Western blot analysis using the specific primary antibodies and HRP-labeled anti-rabbit IgG antibody; (**b**) Expression levels of the key mediators, including Gα, PKC, p-PKC, PI3K and p-PI3K, in the mAChR M2 and M3 signaling pathways, were measured by Western blot analysis using the specific primary antibodies and HRP-labeled anti-rabbit IgG antibody. After determining the intensity of each band using an imaging densitometer, the relative levels of the four proteins were calculated based on the intensity of β-actin. Three to five mice per group were used to prepare the total tissue homogenate, and Western blot analyses were assayed in duplicate in each sample. The data are reported as the mean ± SD. *, *p* < 0.05 compared to the WT group. Abbreviations: WT, Wild type; KO, Knockout type; mAChR, muscarinic acetylcholine receptors; PKC, Protein kinase C; PI3K, Phosphoinositide 3-kinases; HRP, Horseradish peroxidase; IgG, Immunoglobulin G.

Experiments were conducted to determine if the defecation delay in Lep KO mice were accompanied by changes in the regulation of the downstream signaling pathway of mAChRs. The changes in mAChR M2, mAChR M3, Gα, protein kinase C (PKC), p-PKC, phosphoinositide 3-kinases (PI3K), and p-PI3K expression were measured in the transverse colons of a subset of groups. The levels of mAChR M2 and mAChR M3 expression were significantly lower in the Lep KO mice than the WT mice (Figure 8a). However, their downstream signaling pathway exhibited a reverse pattern in the same group. The levels of Gα expression and PKC and PI3K phosphorylation were significantly higher in the Lep KO mice, but the increase rate was varied (Figure 8b). These results show that the defecation delay in Lep KO mice may be associated with the dysregulation of the downstream signaling pathway of mAChRs.

### 2.9. Alterations on the Adipogenesis and Lipolysis in the Transverse Colon of Lep KO Mice

**Figure 9:**
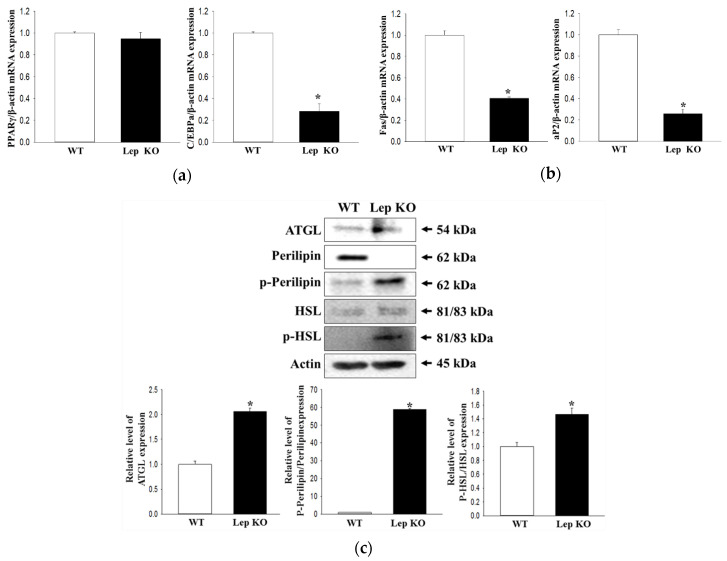
Expression of the lipogenesis and lipolysis associated genes. After purification of the total RNA from the transverse colon, RT-qPCR using the specific primers was performed to analyze the expression levels of the genes associated with adipogenesis (C/EBPα and PPARγ) (**a**) and lipogenesis (Fas and aP2) (**b**). Three to five mice per group were used to prepare total RNA, and RT-PCR were assayed in duplicate for each sample. (**c**) Western blot analysis was performed to detect the phosphorylation or expression of several lipolysis-associated proteins, including perilipin, p-perilipin, HSL, p-HSL, and ATGL. The intensity of each band was determined using an imaging densitometer, and the relative levels of the four proteins were calculated based on the intensity of β-actin. Three to five mice per group were used to prepare the liver tissue homogenate, and Western blot analyses were assayed in duplicate for each sample. The data are reported as the mean ± SD. *, *p* < 0.05 compared to the WT group. Abbreviations: WT, Wild type; KO, Knockout type; RT-qPCR, reverse transcription-quantitative polymerase chain reaction, C/EBP, CCAAT/enhancer-binding protein; PPARγ, Peroxisome proliferator-activated receptor γ; Fas, Fatty acid synthase; aP2, Fatty acid-binding protein; ATGL, Adipose triglyceride lipase; HSL, Hormone-sensitive lipase.

Finally, the expression levels of several key genes that adipogenesis and lipolysis in the transverse colon were measured to elucidate the correlation between constipation and increase in lipid droplet. As shown in Figure 9, significant changes were observed in the mRNA levels of the adipogenesis and lipogenesis-related genes in Lep KO mice except for peroxisome proliferator-activated receptor γ (PPARγ). The mRNA levels of CCAAT/enhancer-binding protein (C/EBP)α for adipogenesis and fatty acid-binding protein (aP2) and fatty acid synthase (FAS) for lipogenesis were significantly lower in the Lep KO mice than the WT mice. On the other hand, the expression of adipose triglyceride lipase (ATGL) and the phosphorylation of perilipine and hormone-sensitive lipase (HSL) were significantly higher in the transverse colon of Lep KO mice (Figure 9c). These results suggest that the alternative response of adipogenesis and lipogenesis in the transverse colon can contribute to the defecation delay in Lep KO mice.

## 3. Discussion

Various animal models have been used in mechanism studies and evaluations of therapeutic drugs for human disease because they show similarity to humans in terms of genetics, anatomy, and physiology [30]. Models are produced by either exposing physicochemical agents, including X-rays and N-ethyl-N-nitrosourea (ENU), or genetic engineering techniques, such as transgenesis, single-gene knockouts, and knock-ins [31]. After production, many models show the expected phenotypes for human disease. Despite this, many unexpected and unexplained phenotypes, including sudden death, reproductive impotence and some chronic diseases, have been observed in these models during or after the onset of the diseases [32,33]. These phenotypes also contribute to the establishment of a novel animal model with human disease-like phenotypes. As part of these studies, an attempt was made to find novel, unexpected phenotypes in CRISPR-Cas9-mediated Lep KO mice with obesity. The results of the present study provide scientific evidence that the key phenotypes for constipation were observed in Lep KO mice from 12 to 24 weeks compared to WT mice. The results of the present study show that constipation detected in Lep KO mice might be linked tightly to dysregulation of the myenteric neuron function, smooth muscle contraction, the mAChR signaling pathway, and lipid metabolism.

Lep KO mice used in previous studies showed a range of obesity phenotypes, including hepatic lipid accumulation, body weight gain, and alterations to the serum lipid profile [14,17]. In addition, they showed metabolic abnormalities in the reproductive capacity, the hypothalamic-pituitary-adrenal (HPA) axis, and the thyroid axis [34,35]. On the other hand, the above phenotypes are affected by the genetic background (C57BL/6J, C57BL/KsL, BALB/cJ, and FVB/N), even though they are common to obesity, hyperphagia, and reduced energy expenditure. C57BL/6J exhibit mild hyperglycemia and an increase in insulin level during 8–12 weeks of age, while C57BL/KsL show hyperglycemia, diabetes, and maximum weight at three to four months of age [36,37]. In particular, the plasma levels of cholesterol were significantly higher in Lep KO mice; a significant elevation is seen in HDL-C rather than very-low-density lipoprotein (VLDL) or LDL-C [38]. This study analyzed various obesity phenotypes to conform to the obese conditions of Lep KO mice with a C57BL/6J background at 24 weeks of age. The LDL-C level was two times higher in Lep KO mice, but the HDL-C level was 2.75 times lower in the same group. Most phenotypes for obesity were similar to previous values. However, some regulating factors for adipogenesis and lipolysis in transverse colon of Lep KO mice were investigated in this study for the first time. These results suggest a correlation between the alternative response for the lipid metabolism and induction of constipation in the transverse colon of Lep KO mice, even though further research in the molecular mechanism for their interaction will be needed.

The constipation phenotypes in an animal model were determined based on several key factors, including the defecation delay, alteration of the stool parameters, and detection of histopathological changes [6,7]. In each GEM model, however, the phenotypical factors for the symptoms of constipation varied widely, and their age was not matched between studies. BNDF +/−mice showed an approximately 39% decrease in stool frequency, 4% stool water content, and 75% gastrointestinal transit in eight-week-old mice, whereas Nrp1^SMKO^ mice exhibited an approximately 80% lower stool number and 27% smaller colon length [6,9]. An approximately 51%−39% decrease in stool number, weight, and water content were detected in eight-week-old A_2B_AR^-/-^ mice [10]. In addition, Tg2576 mice showed a 51–60% decrease in the stool parameters and a 70–78% decrease in the histopathological parameters at 11 months of age [7]. In this study, the stool and histopathological parameters were measured to identify constipation of Lep KO mice at 24 weeks of age. Most parameters were 44–66% (stool parameters) and 25–39% (histopathological parameters) lower in Lep KO mice than WT mice at the same age. Most results from the current study for the constipation phenotypes in Lep KO mice were similar to previous studies that analyzed the constipation phenotypes in the GEM model. In particular, the results first show that the incidence rate of constipation in Lep KO mice was maintained at 70–80% from 12 to 24 weeks. Most studies of the constipated model did not provide any scientific evidence for the incidence of constipation.

The function and regulation of gastrointestinal motility is a complex process mediating the cooperation and communication of multiple cells involving the enteric neurons, ICC, and smooth muscle cells [39]. The number of ICC and the myenteric plexus were significantly lower in the colon and rectum of patients with slow-transit constipation [40,41], where the delay of colon transit and bead expulsion and the decrease in ICC and myenteric plexus were detected in the ascending colon and descending colon of two-year old male Fischer-344 rats [42]. The immunoreactive expression for PGP9.5 was significantly lower in the myenteric and submucosal plexus of BDNF-/- mice with slow gut motility [6]. The lop-induced constipation institute of cancer research (ICR) and Sprague Dawley (SD) model showed a decrease in two ICC markers (C-kit and SCF), an increase in two enteric-nerve-related factors (transient receptor potential vanilloid 1 (TRPV1) and NOS), and a decrease in the other enteric-nerve-related factors (PGP9.5, Brain-derived neurotrophic factor (BDNF), and GNDT) [43,44]. In a few studies, however, the density and distribution of ICC in patients with slow transit constipation were not significantly different from normal colon [45]. Some significant alterations to the distribution and function of myenteric plexus were detected in cloned tissue during diet-induced obesity. Treatment with a high-fat diet for 17 weeks induced neural loss in the myenteric plexus of the distal colon of Swiss mice [46]. High-fat diet-induced obesity for 12 weeks resulted in an increase in the signaling of two key mediators (acetylcholine and serotonin) in the enteric nervous system of distal clone [47]. In contrast, the availability of serotonin and the number of enterochromic cells were lower in the colon of high-fat diet-induced obese rats [48]. This study examined the density and function of nitrergic enteric neurons, myenteric neurons, ICC, and smooth muscle cells to investigate the correlation between defecation delay and GI motility. The expression levels of some markers for enteric neurons, ICC, and smooth muscle cells were lower in the Lep KO mice than WT mice, as shown in Figure 6 and Figure 7, even though the AChE activity was increased. These results are mostly consistent with the previous finding that enteric neurons, ICC, and smooth muscle cells were decreased during constipation. However, this study provides limited information on gastrointestinal motility because two or three key factors for three mediating cells were analyzed in the transverse colon. Therefore, multifactor analyses and mechanism studies will be necessary to clarify the role of the enteric neurons, ICC, and smooth muscle cells in Lep KO mice during constipation.

Meanwhile, this study has some limitations and weak points for leptin role on the experimental design and sample analysis. Firstly, the results of CRISPR-Cas9 leptin KO mice used in this study need to be compared and analyzed in other obesity models, including GEM obesity model with single autosomal recessive mutation of leptin gene [14] and high-fat-diet-induced obese model. However, the differences for microbiome among animal models need not be considered as a major factor because the microbiological condition of the WT and C3 KO mice during the experiment period has been proven by the health-monitoring report. It is also necessary to analyze how leptin control the proliferation and growth of intestinal muscle cell, enteric neurons, paneth cell and goblet cells to verify the correlation between leptin deficiency and molecular mechanism of constipation. Furthermore, a restore test is needed to see if the observed measure of constipation can be reversed by the treatment of leptin.

## 4. Materials and Methods

### 4.1. Animal Study

The animal protocol to characterize novel phenotypes was reviewed and approved by the Pusan National University-Institutional Animal Care and Use Committee (PNU-IACUC) based on the ethical procedures for scientific care (Approval Number PNU-2019–2293). All mice were maintained at the Pusan National University-Laboratory Animal Resources Center, accredited by the Korea Food and Drug Administration (KFDA) (Accredited Unit Number-000231) and the Association for Assessment and Accreditation of Laboratory Animal Care (AAALAC) International (Accredited Unit Number; 001525). All mice were provided access to a standard irradiated chow diet (Samtako BioKorea Inc., Osan, Korea) and water *ad libitum*. Throughout the experiment, the mice were maintained in a specific pathogen-free (SPF) state under a strict light cycle (on at 08:00 h; off at 20:00 h) at 23 ± 2°C and 50 ± 10% relative humidity.

The adult Lep heterogeneous (HT) type of C57BL/6-Lep^em1Shwl^/Korl mice were kindly provided by the Department of Laboratory Animal Resources (Laboratory Animal Resources Bank) in the National Institute of Food and Drug Safety Evaluation (NIFDS, Chungju, Korea). The mice were generated using the CRISPR-Cas9 system, as described previously [21]. The WT and Lep KO mice were produced by mating male Lep HT mice and female Lep HT mice. The Lep KO mice were identified genomically from the DNA extracted from the tails of 1-week-old founder mice through DNA-PCR analysis. Exon 2 of the Lep gene was amplified using two primer sets: 5′- TCC CAG GGA GGA AAA TGT GCT -3′ (forward for Lep), 5′- TGA CAT GTT TCT CAG ACT CTG GTT -3′ (reverse for Lep). Briefly, 10 pmole of sense and antisense primers were added, and the reaction mixture was subjected to 30 cycles of amplification in a thermal cycler (Perkin-Elmer, Waltham, MA, USA) under the following conditions: 30 s at 94 °C, 30 s at 60 °C, and 45 s at 72 °C. After amplification, the product (140 and 219 bp) levels were quantified by 1.2% agarose gel electrophoresis, and the band patterns were detected by Kodak Electrophoresis Documentation (Eastman Kodak, New York, NY, USA) (Figure 1a).

The stool parameters for the constipation phenotypes were analyzed in eight to 24-week-old WT mice (n = 7) and KO mice (n = 7). At 8, 12, 16, 20, and 24 weeks, the total stools were collected from the metabolic cage of each group, and the total number, weight, water content and morphology were measured using the appropriate methods. At 24 weeks, all mice were euthanized using CO_2_ gas, after which the fat, liver, transverse colon, and serum samples were acquired and stored at −70 °C in Eppendorf tubes until assayed.

### 4.2. Measurement of Body and Fat Weight

At 8, 12, 16, 20, and 24 weeks, the bodyweight of WT and Lep KO mice was measured at 9:00 a.m. every Wednesday using an electronic balance (Mettler Toledo, Greifensee, Switzerland), according to the KFDA guidelines. In addition, the weights of retroperitoneal fat collected from sacrificed WT and Lep KO mice, 24 weeks of age, were determined using the same method used to measure the body weight.

### 4.3. Measurement of Food Intake and Water Consumption

The food weight and water volume of WT and Lep KO mice were measured at 24 weeks of age using an electrical balance (for food weight) and a measuring cylinder (for water volume). All measurements were performed two times to ensure accuracy. The average food intake and water consumption were then calculated using the measured data.

### 4.4. Measurement of Stool Parameters

At eight, 12, 16, 20, and 24 weeks, WT and Lep KO mice were bred individually in metabolic cages to provide uncontaminated stool and urine samples (Daejong Instrument Industry Co., LTD, Seoul, Korea). Briefly, the stools excreted from each WT and KO mouse were collected at 9 a.m. every Wednesday and weighed in duplicate using an electric balance. The total number of stools was counted two times per animal, and their morphology was analyzed by taking photographs. The stool moisture content was also estimated by simple calculation method as follows

Stool moisture content = [(A-B)/A] × 100

where A is the weight of fresh stools collected after Lop administration, and B is the weight of stools after drying at 60 °C for 24 h. The incidence rate of constipation was analyzed as follows

Incidence rate = Number of constipated mice/Total number of analyzed mice × 100

### 4.5. Measurement of Gastrointestinal Transit Ratio and Intestinal Length

The gastrointestinal transit ratio was measured using the method described elsewhere [49]. Briefly, 24-week-old WT and Lep KO mice were fasted for 18 h before the experiment, but were given access to water ad libitum. Each mouse in the subset group was fed 1 mL of charcoal meal (3% suspension of activated charcoal in 0.5% aqueous methylcellulose) (Sigma-Aldrich Co., St. Louis, MO, USA). After 30 min of treatment, the mice were euthanized with CO_2_, and the intestinal tract was collected from the abdominal cavity. The intestinal charcoal transit ratio was estimated by simple calculation method as follows

Charcoal transit ratio (%) = [(total small intestine length-transit distance of charcoal meal)/total small intestine length)] × 100

The total intestinal length was also measured from the stomach to the anus in duplicate.

### 4.6. Histopathological Analysis

The liver, fat, and transverse colons collected from WT and Lep KO mice were fixed in 10% formalin for 48 h. The samples were then embedded in paraffin wax, after which they were cut into 4-μm-thick sections and stained with hematoxylin and eosin (H&E, Sigma-Aldrich Co.). The mucosa layer thickness and muscle layer thickness in the transverse colon sections were then analyzed by optical microscopy using the Leica Application Suite (Leica Microsystems, Wetziar, Germany). In addition, the adipocyte size and lipid droplet number were examined microscopically in the fat and liver sections at 400× magnification. Professor Beum Seok Han, a pathologist at the Department of Pharmaceutical Engineering, Hoseo University (Asan), Korea, characterized the pathological features of each tissue.

For detection of nNOS via immunofluorescence staining analysis, transverse colon tissues were fixed in 10% formalin for 48 h, embedded in paraffin blocks, and sliced into 4-µm-thick sections. Sections (n = 5) were then deparaffinized with xylene, rehydrated with different concentrations of EtOH, and pretreated with blocking buffer containing 10% goat serum (Vector Laboratories, Inc., Burlingame, CA, USA) in PBS solution for 30 min at room temperature. The pretreated sections were then incubated with anti-nNOS (Abcam Com., Cambridge, UK) antibodies diluted 1:200 in blocking buffer. After thorough washing in PBS solution, the sections were incubated with goat fluorescein isothiocyanate (FITC)-labeled anti-rabbit IgG (1:200, Thermo Fisher Scientific Inc., Wilmington, MA, USA) for 45 min, washed thrice in PBS for 30 min each, and mounted with a vector shield mounting medium. Finally, green fluorescence intensity on the tissue section of transverse colon were detected using a Motic AE31 Inverted Phase Contrast Fluorescence Microscope (Motic Incoporation Ltd., Causeway Bay, Hong Kong).

Mucin-staining analysis was achieved by fixing the transverse colons collected from the mice of all subset groups in 10% formalin for 48 h, then embedding the samples in paraffin wax and sectioning into 4-μm-thick slices that were then deparaffinized with xylene and rehydrated. The mounted tissue sections were rinsed with distilled water and stained using an Alcian Blue Stain kit (IHC WORLD, Woodstock, MD, USA), and the stained patterns in the transverse colon sections were observed by optical microscopy.

### 4.7. Serum Biochemical Analysis

The serum was obtained for biochemical analysis by centrifuging the whole blood at 1500× *g* for 10 min. The serum biochemical components, including Glu, LDL-C, HDL-C, TG, and TC, were assayed using an automatic serum analyzer (Hitachi 747; Hitachi, Tokyo, Japan). All assays were measured in duplicate using fresh serum.

### 4.8. TEM Analysis

The transverse colon tissues collected from the mice of the subset groups were fixed in a 2.5% glutaraldehyde solution, rinsed with a 1× PBS solution, dehydrated with increasing concentrations of the EtOH solution, post-fixed in 1% osmium tetroxide (OsO_4_) for 1–2 h at room temperature, and embedded in Epon 812 media (Polysciences, Hirschberg an der Bergstrasse, Germany). Ultra-thin sections of the transverse colon tissue (70 nm thick) were placed on holey formvar-carbon coated grids. The grids were subjected to negative staining using uranyl acetate and lead citrate. The ultrastructure and distribution of Lieberkühn crypts in the transverse colon were examined by TEM (Hitachi).

### 4.9. Western Blotting Analysis

The total proteins were collected from the transverse colons of WT and Lep KO mice using the Pro-Prep Protein Extraction Solution (Intron Biotechnology Inc., Seongnam, Korea) according to the manufacturer’s protocol. The acquired proteins were then centrifuged at 13,000 rpm at 4 °C for 5 min, after which the total protein concentrations were determined using a SMARTTM Bicinchoninic Acid Protein assay kit (Thermo Fisher Scientific Inc.). The proteins (30 μg) were subjected to 4%–20% sodium dodecyl sulfate-polyacrylamide gel electrophoresis (SDS-PAGE) for 3 h, and the resolved proteins were transferred to nitrocellulose membranes for 2 h at 40 V. The membranes were then probed overnight with the following primary antibodies at 4 °C: anti-nNOS (Abcam Com.), anti-NSE (Abcam Com.), anti-C-kit (DAKO, Kyoto, Japan), anti-PGP9.5 (Abcam Com.), anti-MLC (Abcam Com.), anti-p-MLC (Abcam Com.), anti-HSL (Cell Signaling Technology Inc., Danvers, MA, USA), anti-p-HSL (Cell Signaling Technology Inc.), anti-perilipin (Cell Signaling Technology Inc.), anti-p-perilipin (Cell Signaling Technology Inc.), anti-ATGL (Cell Signaling Technology Inc.), anti-Gα (Abcam Com.), anti-mAChR M2 (Alomone Labs, Jerusalem, Israel), anti-mAChR M3 (Alomone Labs), anti-PKC (Cell Signaling Technology Inc.), anti-p-PKC (Cell Signaling Technology Inc.), anti-PI-3K (Cell Signaling Technology Inc.), anti-p-PI3K (Cell Signaling Technology Inc.), or anti-actin (Sigma-Aldrich Co.). The membranes were then washed with a washing buffer (137 mM NaCl, 2.7 mM KCl, 10 mM Na_2_HPO_4_, 2 mM KH_2_PO_4_, and 0.05% Tween 20), followed by incubation with 1:1000 diluted horseradish peroxidase-conjugated goat anti-rabbit IgG (Zymed Laboratories, South San Francisco, CA, USA) for 2 h at room temperature. The blots were then developed using a Chemiluminescence Reagent Plus kit (Pfizer Inc., Gladstone, NJ, USA). Signal images of each protein were then acquired using a digital camera (1.92 MP resolution) of the FluorChem^®^ FC2 Imaging system (Alpha Innotech Corporation, San Leandro, CA, USA). The protein densities were semi-quantified using the AlphaView Program, version 3.2.2 (Cell Biosciences Inc., Santa Clara, CA, USA).

### 4.10. Quantitative Real-Time-Polymerase Chain Reaction (RT-qPCR) Analysis

The frozen transverse colon tissue was chopped with scissors and homogenized in an RNA Bee solution (Tet-Test, Friendswood, TX, USA). The total RNA molecules were isolated by centrifugation at 15,000 rpm for 15 min, after which RNA concentration was measured using a NanoDrop Spectrophotometer (Allsheng, Hangzhou, China). The expression of the SOD gene was measured by annealing the total RNA (5 µg) from the liver tissue with 500 ng of oligo-dT primer (Thermo Fisher Scientific Inc.) at 70 °C for 10 min. The complementary DNA (cDNA) was synthesized using the Invitrogen Superscript II reverse transcriptase (Thermo Fisher Scientific Inc.). qPCR was performed using the cDNA template obtained (2 µL) and 2× Power SYBR Green (6 µL; Toyobo Life Science, Osaka, Japan) containing the specific primers as follows: C/EBPα sense primer 5′-GAGCT GAGTG AGGCT CTCAT TCT-3′ and antisense primer 5′-TGGGA GGCAG ACGAA AAAAC-3′; PPARγ sense primer 5′- GCCCA CCAAC TTCGG AATC-3′ and antisense primer 5′- TGCGA GTGGT CTTCC ATCAC-3′; FAS sense primer 5′-AAGTG TCTGG ACTGT GTCAT TTTTA CA-3′ and antisense primer 5′- TTAAT TGTGG GATCA GGAGA GCAT-3′; aP2 sense primer 5′- TGGCC AAGCC CAACA TG -3′ and antisense primer 5′-TCATC AGCGT AAATG G-3′; AQP3 sense primer 5′-GGTGG TCCTG GTCAT TGGAA-3′ and antisense primer 5′-TCAAC CCTGC CCGTG ACT’;AQP8 sense primer 5′-GTAGT ATGGA CCTAC GTGAG ATCAA GG-3′ and antisense primer 5′-AGAAC CTTTC CTCTG GACTC ACCAC C-3′; MUC2 sense primer 5′-GCTGC TCATT GAGAA GAACG ATGC-3′ and antisense primer 5′-CTCTC CAGGT ACACC ATGTT ACCAG G-3′; β-actin sense and antisense primers 5′- ACCAGTTCGCCATGGATGAC-3′ and 5′- ATATCGCTGCGCTGGT-3′. The thermal cycling conditions for DNA amplification were consisted of Holding stage (as 1 min at 95 °C), Cycling stage (40 cycles of 15 s at 95 °C, 15 s at 57 °C, and 45 s at 72 °C), and Melt curve stage (15 s at 95 °C and 1 min s at 60 °C). The fluorescence intensity was measured at the end of the extension phase of each cycle. The threshold value for the fluorescence intensities of all samples was set manually. The reaction cycle at which the PCR products exceeded this fluorescence intensity threshold during the exponential phase of PCR amplification was considered the threshold cycle (*C*t). Expression of the target gene was quantified relative to that of the housekeeping gene β-actin based on a comparison of the *C*ts at a constant fluorescence intensity using the Livak and Schmittgen’s method [50].

### 4.11. Measurement of GI Hormone Concentrations

The concentration of CCK and gastrin were quantified using an ELISA kit (Cusabio Biotech Co., Ltd., Wuhan, China) according to the manufacturer’s instructions. Briefly, the transverse colon tissues (100 mg) were homogenized in ice-cold 1× PBS (pH 7.2–7.4) using a glass homogenizer (Sigma-Aldrich Co.). The resulting tissue lysates were centrifuged at 1000× *g* for 5 min at 4 °C, after which the supernatant was collected for analysis. After adding the three specific hormone antibodies (separately in each well), the supernatant was incubated for 1 h at 37 °C, to which an HRP-Streptavidin solution was then added and incubated further for 1 h at 37 °C. This was followed by the addition of the TMP One-Step Substrate Reagent followed by incubating a mixture for 30 min at 37 °C. The reaction was quenched by adding the stop solution. Finally, the absorbance of the reaction mixture was read at 450 nm using the Molecular Devices VersaMax Plate Reader (Sunnyvale, CA, USA).

### 4.12. Statistical Analysis

Statistical significance was evaluated using the One-way Analysis of Variance (ANOVA) (SPSS for Windows, Release 10.10, Standard Version, Chicago, IL, USA) followed by a Tukey post hoc *t-*test for multiple comparisons. All values are expressed as the means ± SD. A *p*-value (*p* < 0.05) was considered statistically significant.

## 5. Conclusions

Overall, the study results suggest that Lep-deficiency-induced obesity can induce constipation phenotypes, including a decrease in stool parameters, delay of gastrointestinal transit, alteration of the histopathological structure of the transverse colon, and suppression of mucin in Lep KO mice. In particular, these results provide evidence that the constipation of Lep KO mice is tightly correlated with the dysregulation of gastrointestinal motility-mediating enteric neurons, ICC, and smooth muscle cells. Furthermore, these findings suggest that Lep KO mice are a potential animal model for constipation to evaluate a therapeutic drug and study the molecular mechanism for chronic constipation.

## Supplementary Materials

The following are available online at https://www.mdpi.com/1422-0067/21/24/9464/s1, Figure S1: Bodyweight and feeding behavior of Lep KO mice.

## Abbreviations

GEM	genetically engineered mice
Lep	leptin
KO	knockout
ICC	intestinal Cajal cells
GI	gastrointestinal
mAChRs	muscarinic acetylcholine receptors
PTHS	Pitt-Hopkins syndrome
A2BAR	adenosine 2B receptor
ER	endoplasmic reticulum
AD	Alzheimer’s Disease
TZDs	thiazolidinediones
WT	wild type
H&E	hematoxylin and eosin
Glu	glucose
LDL-C	low-density lipoprotein-cholesterol
HDL-C	high-density lipoprotein-cholesterol
TG	triglyceride
TC	total cholesterol
TEM	tansmission electron microscopy
MUC2	mucin 2
AQP	aquaporin
nNOS	neuronal nitric oxide synthase
NSE	neuron-specific enolase
AChE	acetylcholinesterase
C-kit	Receptor tyrosine kinase
PGP9.5	protein gene product 9.5
MLC	Myosin light chain
CCK	cholecystokinin
PKC	protein kinase C
PI3K	phosphoinositide 3-kinases
PPARγ	peroxisome proliferator-activated receptor γ
C/EBP	CCAAT/enhancer-binding protein
aP2	fatty acid-binding protein
FAS	fatty acid synthase
ATGL	adipose triglyceride lipase
HSL	hormone-sensitive lipase
ENU	N-ethyl-N-nitrosourea
HPA	hypothalamic-pituitary-adrenal
VLDL	very-low-density lipoprotein
ICR	institute of cancer research
SD	Sprague Dawley
TRPV1	transient receptor potential vanilloid 1
BDNF	Brain-derived neurotrophic factor
cDNA	complementary DNA
