# Supplementary material for: Molecular Characterization of Constipation Disease as Novel Phenotypes in CRISPR-Cas9-Generated Leptin Knockout Mice with Obesity
Source: Int J Mol Sci. 2020 Dec 12;21(24):9464. doi: 10.3390/ijms21249464 (PMC7763920; doi:10.3390/ijms21249464)
Supplement: Supplementary file 1 [file ijms-21-09464-s001.pdf]

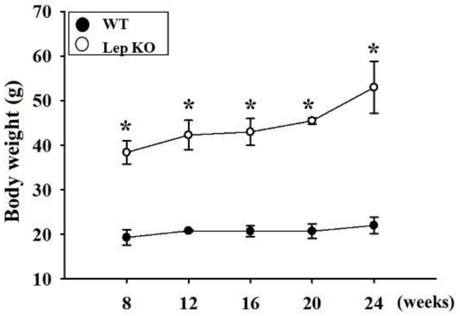

(**a**)

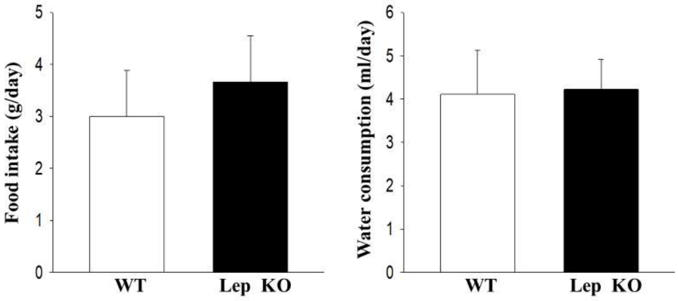

(**b**)

Figure S1. Bodyweight and feeding behavior of Lep KO mice. (**a**) Body weights of the WT and Lep KO mice were measured from eight to 24 weeks, as described in the Materials and Methods. Five to six mice per group were selected from each group, and their weights were assayed in duplicate for each sample. (**b**) Food intake and water consumption were also calculated using the amount of feed (water) supplied and the amount of feed (water) remaining at 24 weeks of age. Five to six mice per group were selected from each group, and the food weights and water volume were measured in duplicate for each sample. The data are reported as the mean ± SD. *, $p < 0.05$ compared to the WT group. Abbreviations: WT, Wild type; KO, Knockout type.
